# High level dynein impairs mitochondrial distribution and differentiation of rhabdomyosarcoma cells

**DOI:** 10.1016/j.isci.2026.116057

**Published:** 2026-05-22

**Authors:** Ting-Ling Ke, Linyi Chen

**Affiliations:** 1 Institute of Molecular Medicine, National Tsing Hua University, No. 101, Section 2, Kuang-Fu Road, Hsinchu 30013, Taiwan; 2 Department of Medical Science, National Tsing Hua University, No. 101, Section 2, Kuang-Fu Road, Hsinchu 30013, Taiwan

**Keywords:** Molecular biology, Cell biology, Specialized functions of cells

## Abstract

Rare disease rhabdomyosarcoma-derived RD cells and RH30 cells are defective in myogenesis. In this study, we demonstrate that mitochondria in these cells are enlarged and display a perinuclear distribution. Given that impaired mitochondrial morphology, trafficking, and activity are implicated in many human diseases, characterizing the link between these phenotypes and their physiological outcomes is essential. We found that RD cells had reduced levels of the myosin motor MYO19 and elevated levels of the dynein motor and MIRO1/2 adaptors. Our findings indicate that impaired local actin-based anterograde transport, together with enhanced microtubule-based retrograde transport, drives this perinuclear mitochondrial clustering. Overexpression of MYO19 in RD cells partially rescued this phenotype, while dynein inhibition altered mitochondrial distribution and restored myogenic differentiation in both RD and RH30 cells. Collectively, these findings reveal an intricate interplay among mitochondrial morphology, distribution, and myoblast fusion that underlies both normal physiology and disease.

## Highlights

• Mitochondria in human rhabdomyosarcoma (RMS) cells cluster at perinuclear region
• RMS cells exhibit reduced myosin motor MYO19 and impaired actin-based anterograde
• High level dynein in RMS cells increase retrograde transport of mitochondria
• Inhibiting dynein rescues myogenic differentiation defect of RMS cells

## Graphical abstract

**Figure:**
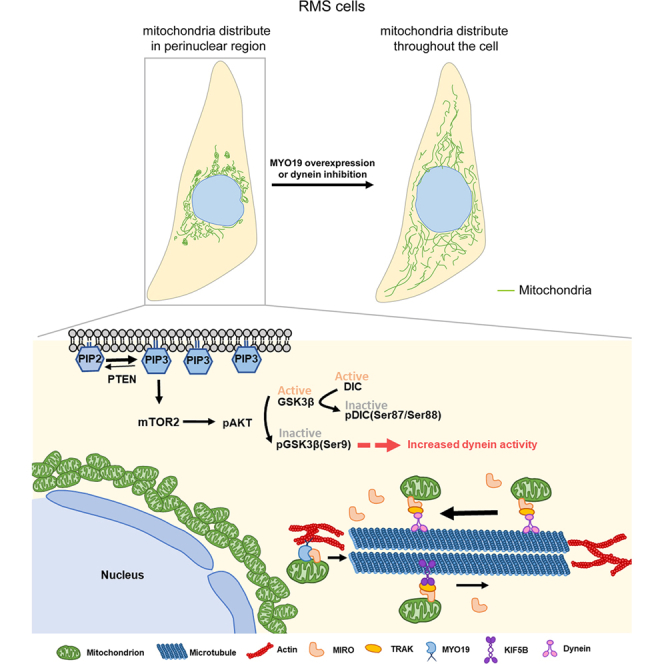

## Introduction

Rhabdomyosarcoma (RMS) accounts for 3% of cancers in children and young adults and leads to early death. The 5-year event-free survival rate of patients with metastatic RMS is only 17.3%.^1^ Two major subtypes of RMS, namely embryonal RMS (eRMS) and alveolar RMS (aRMS) were used to characterize defects in myoblast differentiation and fusion.^2^ Our previous work has characterized these myogenic defects in human eRMS-derived RD cells^3^ as well as the epigenetic regulation of the underlying transcriptional machinery.^4^ In our earlier research, we demonstrated that RD cells exhibit upregulated phosphoinositide-3-kinase (PI3K)-serine-threonine protein kinase (AKT, also called protein kinase B) signaling and downregulated phosphatase and tensin homolog (PTEN) compared to normal C2C12 myoblasts.^3^ Furthermore, time-lapse imaging revealed elevated levels of phosphatidylinositol (3,4,5)-triphosphate (PIP3) at the plasma membrane, which induces distinct morphodynamic changes of plasma membrane that inhibit myoblast fusion.^3^

Beyond plasma membrane dynamics, we are interested in changes in membranous organelles in this system. To meet metabolic demands, membranous organelles such as lysosomes, mitochondria, and nuclei are organized and transported within the cell via a system of motor and adaptor proteins that utilize the cytoskeleton. Mitochondrial positioning is governed by a coordinated system of short-range, actin-based transport, and long-range, microtubule-based transport.^5^ Bidirectional mitochondrial transport along microtubules primarily involves the motor proteins kinesin and dynein.^6^^,^^7^^,^^8^ Adaptor protein small mitochondrial Rho GTPases (MIROs) coordinate with adaptor proteins TRAK1 and TRAK2 to recruit kinesin and dynein to mitochondria, thereby regulating anterograde and retrograde mitochondrial trafficking on microtubules.^9^^,^^10^^,^^11^ Among the kinesin superfamily proteins (KIFs), KIF5 (kinesin-1) is the primary motor responsible for anterograde transport of mitochondria. While KIF5B is expressed ubiquitously, KIF5A and KIF5C are neuronal-specific. Conversely, retrograde transport on microtubules is predominantly controlled by dynein, and dynein dysfunction is frequently linked to aberrant mitochondrial distribution.^8^ On actin filaments, myosins govern anterograde transport, with MYO19 acting as the dominant motor for actin-based mitochondrial movement.^12^ MIRO1 and MIRO2 serve as essential mitochondrial receptors that recruit MYO19, dynein, or kinesin to coordinate these cytoskeletal movements.^13^ In this study, we discovered that mitochondria in RD and RH30 cells exhibit aberrant morphology and distribution, which correlates with defective myoblast differentiation. By analyzing the distinct perinuclear distribution of mitochondria in these cells, we elucidated the regulatory machinery governing the actin- and microtubule-based mitochondrial transport pathways.

## Results

### Mitochondria are enlarged and aggregated in the perinuclear region of RD and RH30 cells

**Figure 1:**
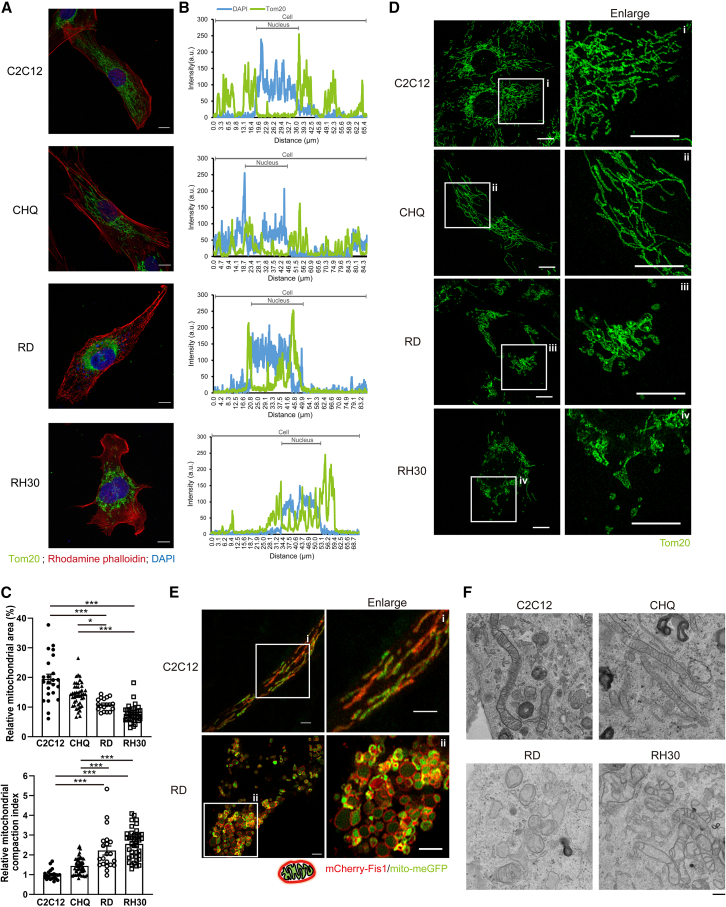
Aggregates of enlarged mitochondria are distributed to the perinuclear region in RMS cell lines (A) Immunofluorescence staining of RMS and control myoblasts using anti-Tom20 (a mitochondrial marker), rhodamine phalloidin (an actin marker) and DAPI (a nuclear marker). Scale bars, 10 μm. (B) Intensity histograms representing quantification of DAPI (blue) and Tom20 (green). (C) Quantification of mitochondrial distribution using ImageJ. The upper image shows relative mitochondrial area quantified as (mitochondrial area/cellular area) ×100%. The lower image shows the mitochondrial compaction index (CI), which was calculated as described in STAR Methods from measured values of mitochondrial area (*A*) and perimeter (*P*). CI values were normalized to average mitochondrial CI of C2C12 cells. Data are presented as mean ± S.E.M. ∗*p* < 0.05, ∗∗∗*p* < 0.001, one-way ANOVA. (D) Immunofluorescence staining using anti-Tom20. Images on the right are enlarged view of the boxed areas. Scale bars, 10 μm. (E) Images of C2C12 and RD cells transfected with mito-meGFP (labeling mitochondrial matrix) and mCherry-Fis1 (labeling mitochondrial outer membrane) constructs for 48 h. Images on the right are enlarged view of the boxed areas. Scale bars, 2 μm. Images in (A), (D), and (E) were taken using a Zeiss LSM-800 confocal microscope. Images in (E) were taken using Airyscan microscopy for super-resolution imaging. (F) The transmission electron micrographs of mitochondrial morphology in cells were taken by Hitachi HT7700 high contrast transmission electron microscope. Scale bars, 500 nm.

Mitochondrial function and adaptive metabolism are critical to myogenesis and muscle maintenance. During myogenic differentiation, mitochondrial mass/volume, respiration, and mtDNA copy number increase markedly.^14^ Conversely, inhibition of mitochondrial activity or reduced mtDNA replication can lead to myogenic defects.^15^^,^^16^ We previously characterized a myogenesis defect in human eRMS-derived RD cells during myoblast fusion.^3^ This study aimed to investigate the role of mitochondria in the myogenic defects of eRMS-derived RD cells and aRMS-derived RH30 cells, using normal mouse C2C12 myoblasts and human CHQ myoblasts as controls. To this end, we first examined the distribution and morphology of mitochondria by immunofluorescence staining using an antibody specific for Tom20 (a mitochondrial marker) and confocal microscopy. Immunofluorescence images and histogram intensities showed that mitochondria were distributed throughout the cell in C2C12 and CHQ cells but were clustered in the perinuclear region in RD and RH30 cells (Figures 1A and 1B). Quantification of the relative area of mitochondrial distribution (calculated by ImageJ as [mitochondrial area/cellular area] × 100%) showed that the mitochondria in RD and RH30 cells were restricted to a significantly smaller area compared to C2C12 and CHQ cells (Figure 1C, top). The mitochondrial compaction index (CI = [2π × (*A*/π)ˆ(½)]/*P*, where *A* and *P* are area and perimeter of mitochondria) was used to indicate the degree of compaction, with higher value representing more localized/clustered distribution of mitochondria. The analysis showed a higher degree of mitochondrial compaction (higher CI) in RD and RH30 cells compared to control cells (Figure 1C, bottom). The two control cell lines, C2C12 and CHQ cells showed similar mitochondrial distribution and compaction index. These data revealed a clear change of mitochondrial distribution in RMS cells compared to normal myoblasts.

In addition to altered distribution, RMS cells displayed distinct morphological changes. While mitochondria appeared predominantly tubular in C2C12 and CHQ cells, they were enlarged and exhibited a “donut-shaped” morphology in RD and RH30 cells (Figure 1D). To distinguish whether the mitochondrial puncta were individual swollen mitochondria or aggregated/fused mitochondria, structures of mitochondria within C2C12 and RD cells were assessed via confocal microscope with Airyscan. Cells were co-transfected with plasmids encoding mCherry-Fis1 (mitochondrial outer membrane marker) and mito-meGFP (mitochondrial matrix marker). Imaging performed 48 h post-transfection allowed for the overlay of these signals to resolve the internal structure. As shown in Figure 1E, the mitochondria in RD cells were enlarged and distinct from the tubular, fused networks observed in control cells. Rather than forming a network, individual mitochondria appeared as singlet structures clustered together. Furthermore, transmission electron microscopy (TEM) was used to provide ultrastructure of mitochondria at a higher magnification and resolution (Figure 1F). In line with the confocal images, expectation maximization (EM) images showed tubular mitochondria with intact mitochondrial cristae in C2C12 and CHQ cells, whereas round-shaped mitochondria with disrupted cristae were present in RD and RH30 cells.

### Clustered mitochondria affect membrane potential and respiratory function

**Figure 2:**
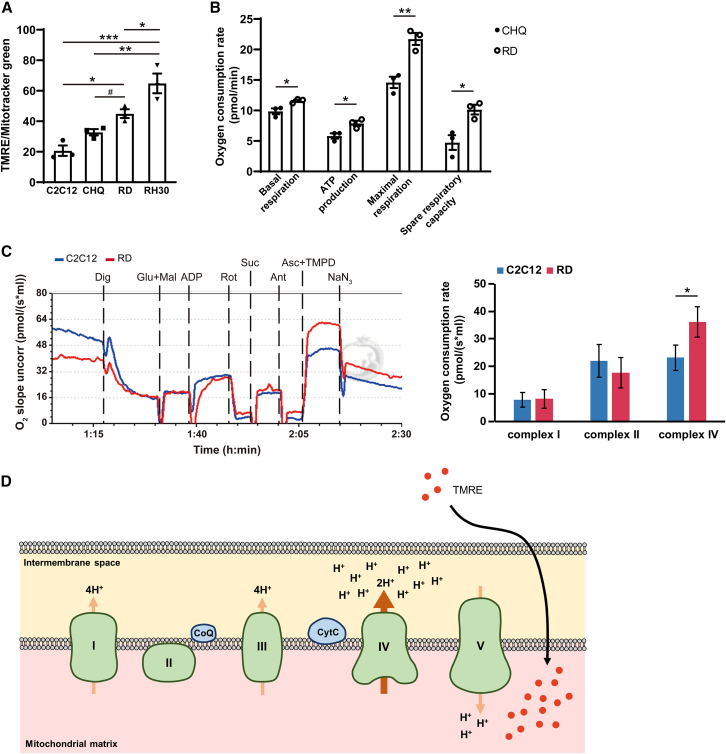
RD cells have a higher mitochondrial membrane potential and high oxygen consumption rate (A) Cells stained with TMRE and MitoTracker green were analyzed by flow cytometry at GM. The ratio of TMRE to MitoTracker Green was used as a measure of relative mitochondrial membrane potential. (B and C) Undifferentiated cells were harvested in growth medium and oxygen consumption was analyzed using Oroboros Oxygraph-2k. (D) Model by which elevated activity of mitochondrial complex IV in RD cells leads to increased proton accumulation in the intermembrane space. A higher proton gradient increases entry of TMRE into the mitochondrial matrix. Data are presented as mean ± SEM; ∗*p* < 0.05, ∗∗*p* < 0.01, ∗∗∗*p* < 0.001, one-way ANOVA. #*p* < 0.05, unpaired *t* test.

While mitochondrial morphogenesis is known to regulate mitochondrial activity, the specific role of mitochondrial distribution on the functional outputs remains unclear. To examine this, we compared mitochondrial membrane potential and relative respiration rate in the cells. In general, higher mitochondrial membrane potential reflects increased mitochondrial activity.^17^ Mitochondrial membrane potential was determined via flow cytometry using tetramethylrhodamine, ethyl ester, perchlorate (TMRE) and MitoTracker green in live cells. The results are expressed as a ratio of TMRE to MitoTracker green to normalize membrane potential against mitochondrial mass. Compared to control cells, RD and RH30 cells displayed a higher TMRE/MitoTracker green ratio, suggesting elevated mitochondrial membrane potential (Figure 2A). Relative mitochondrial respiration was assessed by measuring the oxygen consumption rate using an Oroboros Oxygraph-2k. As shown in Figure 2B, the basal respiration, ATP production, maximal respiration and spare maximal respiration capacity were all higher in RD cells compared to those in CHQ cells, consistent with the elevated mitochondrial membrane potential observed. These findings suggest an increased metabolic activity and a strong capacity to respond to sudden increases in energy demand for RD cells. Comparing the activity of each complex within the electron transport chain, the activity of complex IV was higher in RD cells than that in C2C12 cells (Figure 2C). Complex IV functions by pumping protons from the matrix into the intermembrane space, thereby establishing the proton gradient necessary for Complex V ATP synthase to generate ATP. These results suggested that increased activity of mitochondrial complex IV in RD cells established a high proton gradient, leading to both a higher mitochondrial membrane potential and an increased capacity for ATP generation (Figure 2D).

### Disruption of actin-based MYO19-mediated anterograde transport in RD cells leads to perinuclear distribution of mitochondria

**Figure 3:**
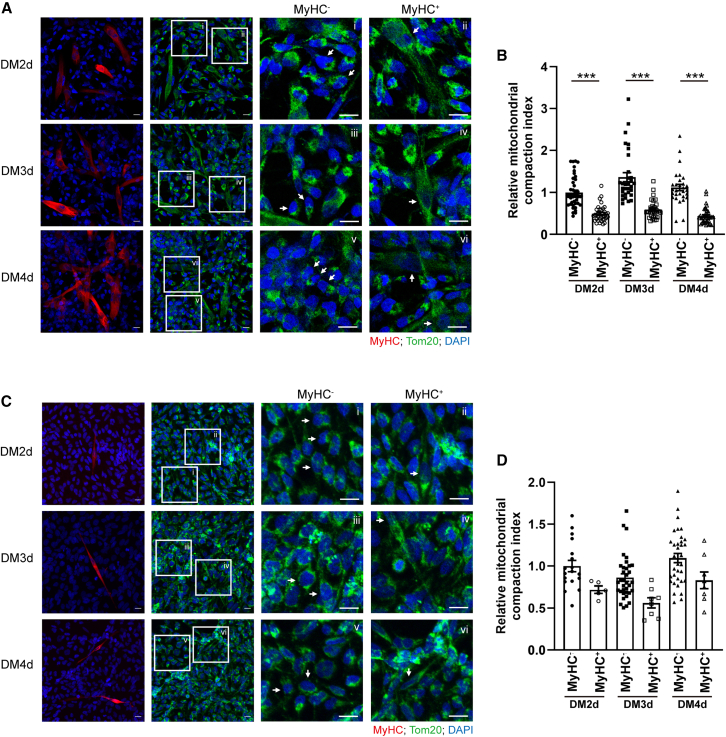
Lower mitochondrial CI in differentiated RD and RH30 cells (A and C) RD cells (A) and RH30 cells (C) were seeded in 12-well plates at 90% confluency and induced cell differentiation with DM medium followed by immunofluorescence staining using anti-MyHC (red) and Tom20 (green) antibodies on 2 days after differentiation induction (DM2d) to DM4d. DAPI (blue) was used to indicate nuclei. Images were taken on an LSM-800 confocal microscope. Images on the right are enlarged from the boxed areas showing MyHC^−^ or MyHC^+^ cells. The white arrows indicate the MyHC^−^ or MyHC^+^ cells. Scale bars, 20 μm. (B and D) Mitochondrial CI of MyHC^+^ and MyHC^−^ cells normalized to the average CI value of MyHC^−^ cells at DM2d. Data are presented as mean ± SEM; ∗∗∗*p* < 0.001, one-way ANOVA (*n* = 5–44).

**Figure 4:**
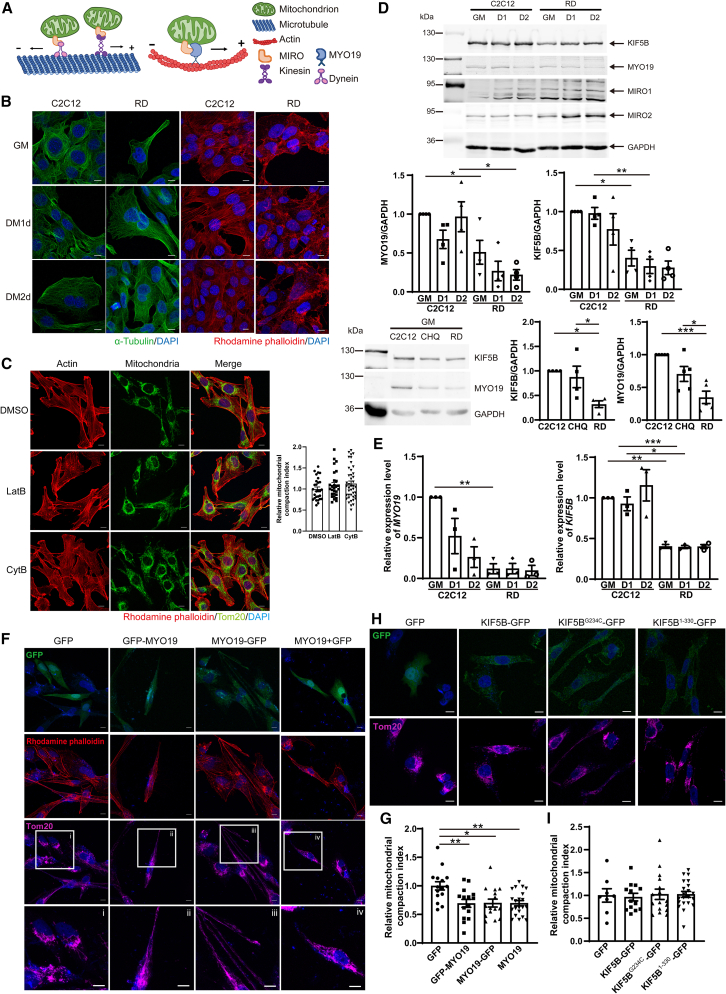
Overexpression of MYO19 rescues mitochondrial distribution in RD cells (A) Mitochondrial trafficking on microtubules and actin is regulated by adaptor protein MIRO and three different motor proteins. On microtubules, dynein is responsible for retrograde transport and kinesin is responsible for anterograde transport. On actin filaments, anterograde transport is regulated by MYO19. (B) C2C12 cells and RD cells were fixed and immunostained with anti-tubulin (green) or rhodamine phalloidin (red) with DAPI (blue) to label nuclei. Images were taken on an LSM-800 confocal microscope. Scale bars, 10 μm. (C) C2C12 cells were treatment with DMSO (vehicle control), 300 nM latrunculin B (Lat B) or 600 nM cytochalasin B (Cyt B) for 24 h followed by immunostaining with anti-Tom20, rhodamine phalloidin, and DAPI. Images were taken on an LSM-800 confocal microscope. Scale bars, 10 μm. Mitochondrial CI were calculated and showed on the right image. (D) Levels of MYO19, KIF5B, MIRO1, and MIRO2 were analyzed by western blotting in CHQ, C2C12, and RD cells. Protein levels were normalized to GAPDH. GM, growth medium; D1, 1 day after differentiation induction. (E) *MYO19* and *KIF5B* expression was analyzed via semi-quantitative PCR in C2C12 and RD cells. Data were normalized to *GAPDH* expression. (F and H) RD cells were transfected with the indicated plasmids for 48 h, followed by immunofluorescence staining using anti-Tom20 and rhodamine phalloidin. Images were taken on an LSM-800 confocal microscope. Scale bars, 10 μm. (G and I) Mitochondrial CI was analyzed in transfected cells was normalized to average CI value of GFP control cells. At least 30 cells were quantified in each group. In (C), (D), (E), (G), and (I), data are presented as mean ± SEM; ∗*p* < 0.05, ∗∗*p* < 0.01, ∗∗∗*p* < 0.001, one-way ANOVA.

Previous studies have reported the differentiation defect of RD cells and RH30 cells.^3^^,^^18^ To determine the relationship between mitochondrial distribution and myogenic differentiation, we used myosin heavy chain (MyHC) as a myogenic differentiation marker and Tom20 as a mitochondrial marker. We assessed the distribution of mitochondria in undifferentiated (MyHC^−^) and differentiated (MyHC^+^) cells from 2 days after differentiation (DM2d) to DM4d (Figures 3A and 3C). The images showed the signal of differentiation marker MyHC (red) and mitochondria (green). The boxed regions indicated the MyHC^−^ or MyHC^+^ cells, which were enlarged on the right images in Figures 3A and 3C. These images revealed that in MyHC^−^ cells, mitochondria were primarily clustered in the perinuclear region. In contrast, MyHC^+^ cells exhibited mitochondria distributed throughout the cytoplasm. Then the mitochondrial CI of MyHC^+^ and MyHC^−^ cells were calculated. Mitochondrial CI was lower in MyHC^+^ RD cells compared to that in MyHC^−^ cells during DM2d-DM4d, indicating that differentiation correlates with a more peripheral mitochondrial distribution (Figure 3B). The fact that no significant difference was observed between MyHC^+^ and MyHC^−^ RH30 cells is likely due to extremely low differentiation rate of RH30 cells (approximately 0.5%). Nonetheless, a trend of reduced CI in MyHC^+^ cells compared to MyHC^−^ cells was still evident (Figure 3D). These results suggest that successful myoblast differentiation is associated with the translocation of mitochondria toward the cell periphery. Based on this result, we hypothesized that the perinuclear accumulation of mitochondria observed in RD and RH30 cells contributes to their impaired myogenic differentiation. Along this line, the transport of mitochondria to the plasma membrane and leading edge of cells has previously been associated with the increased proliferation and invasion of cancer cells.^19^^,^^20^ Thus, the reduced peripheral localization of mitochondria in RD and RH30 cells might be expected to correlate with decreased proliferation and enhanced differentiation, compared to normal myoblasts C2C12 or CHQ cells. However, contrary to this expectation, RD cells exhibited defects in myoblast differentiation and fusion.^3^ These findings suggest that mitochondrial trafficking to cell periphery may be essential for proper myoblast fusion. Mitochondria not only supply energy but also regulate calcium buffering within cells, and thus their cellular distribution likely impacts physiological functions and cell fate determination during development. The distribution of mitochondria is controlled primarily by bidirectional trafficking on the cytoskeleton,^21^ which requires dedicated coordination of transport via microtubules and actin filaments. Long-range mitochondrial transport is mainly facilitated by the coupling of mitochondria to microtubule motors such as kinesins and dynein. Shorter-range mitochondrial transport involves the actin cytoskeleton and its associated myosin motors, particularly MYO19^22^^,^^23^ (Figure 4A). To test whether the perinuclear aggregation in RD cells resulted from dysregulated trafficking, we first examined the morphology of microtubules and actin filaments in C2C12 and RD cells. Microtubule network structure was not obviously different between C2C12 and RD cells. In contrast, while actin bundles in C2C12 cells formed stress fibers, RD cells exhibited a severe defect in actin bundle structure with some random fragmented actin filaments (Figure 4B). To determine if this actin defect was the primary driver of mitochondrial clustering, C2C12 cells were treated with actin inhibitor latrunculin B and cytochalasin B. Figure 4C showed mitochondrial CIs were slightly increased with no statistical difference following actin disruption. This finding suggests that while actin bundles are severely defective in RD cells, this defect is not the primary driver of perinuclear mitochondrial aggregation.

To better understand the mechanism that regulates the trafficking and distribution of mitochondria in RD cells, we next examine expression levels of proteins and genes involved in mitochondrial anterograde transport. We found that the actin-based motor protein MYO19 was significantly downregulated in RD cells compared to C2C12 and CHQ cells at both the protein and gene levels (Figures 4D and 4E). We then investigated whether restoring MYO19 could rescue the perinuclear distribution phenotype. To exclude the possible effect of GFP tag on motor activity of MYO19, we constructed N-terminally-tagged GFP-MYO19, C-terminally-tagged MYO19-GFP, and untagged full-length MYO19 constructs. RD cells were transiently transfected with GFP, GFP-MYO19, MYO19-GFP, or MYO19 constructs for 48 h. Immunofluorescence images using anti-Tom20 and rhodamine phalloidin showed that mitochondria were distributed more evenly in RD cells transfected with the GFP-MYO19, MYO19-GFP and MYO19 constructs compared to the GFP control (Figure 4F). Consistently, the mitochondrial CI was significantly lower in RD cells expressing MYO19 constructs (Figure 4G). These data indicated that increasing actin-based MYO19-dependent anterograde mitochondrial trafficking could partially reverse the perinuclear mitochondrial distribution in RD cells.

Both protein levels and gene expression of the microtubule-based motor KIF5B were significantly lower in RD cells compared to C2C12 or CHQ cells (Figures 4D and 4E). To assess the role of microtubule-based transport, KIF5B was overexpressed in RD cells. RD cells were transiently transfected with a GFP, KIF5B-GFP, KIF5B^G234C^-GFP (deficient in ATP hydrolysis), or KIF5B^1−330^-GFP (lacking the cargo-binding domain) constructs for 48 h followed by immunofluorescence staining with anti-Tom20. In this case, the mitochondria remained in the perinuclear region as assessed by both imaging and CI values (Figures 4H and 4I). We also analyzed levels of the adaptor proteins MIRO1 and MIRO2, which are responsible for coordinating trafficking between microtubules and actin filaments. We found that both MIROs were higher in RD cells than in C2C12 cells (Figure 4D). Elevated levels of MIROs suggest a compensatory response to the reduced levels of the actin- and microtubule-based motors MYO19 and KIF5B in RD cells. Collectively, these data demonstrated that the perinuclear distribution of mitochondria in RD cells could be attributed primarily to a defect in actin-based MYO19-dependent anterograde mitochondrial trafficking.

### Dynein-mediated retrograde transport also contributes to the perinuclear mitochondrial distribution in RD cells

**Figure 5:**
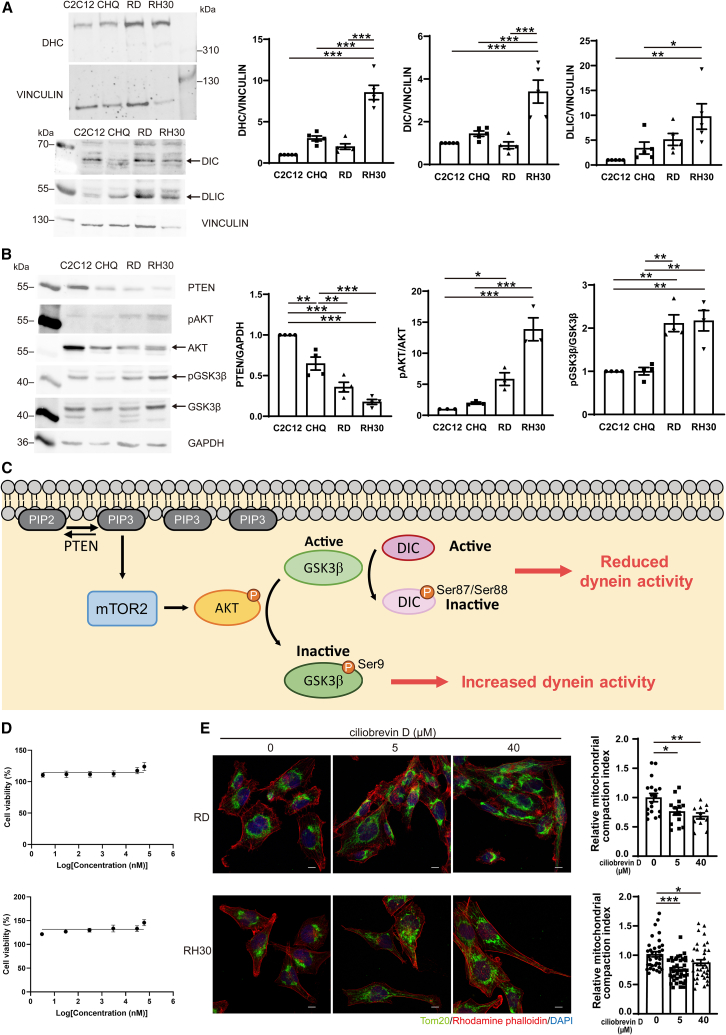
Inhibition of dynein reduces the perinuclear distribution of mitochondria in RD cells (A and B) Levels of DHC, DIC, DLIC, PTEN, pAKT, AKT, pGSK3β, and GSK3β were analyzed by western blotting in CHQ, C2C12, RD, and RH30 cells. DHC, DIC, and DLIC were normalized to VINCULIN. PTEN was normalized to GAPDH. Data from at least three independent experiments are presented as mean ± SEM; ∗*p* < 0.05, ∗∗*p* < 0.01, ∗∗∗*p* < 0.001, one-way ANOVA. (C) In RMS cells, elevated PIP3 in the plasma membrane activates the mTORC2/AKT pathway. Phospho-AKT phosphorylates GSK3β at Ser9 and inactivates it. Inactivation of GSK3β would lead to reduced phosphorylation of DIC, resulting in higher dynein activity. (D) RD cells (top) and RH30 cells (bottom) were treated with 3 nM–60 μM ciliobrevin D for 24 h. Cell viability was measured using CellTiter-Glo reagent. (E) RD cells (top) and RH30 cells (bottom) were treated with 5, 10, or 40 μM ciliobrevin D for 2 h followed by immunostaining with anti-Tom 20 antibody, rhodamine phalloidin and DAPI. Images were taken on an LSM-800 confocal microscope. Scale bars, 10 μm. Mitochondrial CIs of RD cells (top) and RH30 cells (bottom) were calculated and normalized to the average CI value of the untreated control. Data are presented as mean ± SEM; ∗*p* < 0.05, ∗∗*p* < 0.01, ∗∗∗*p* < 0.001, one-way ANOVA.

Because mitochondrial distribution is regulated by bidirectional transport, we next investigated whether dynein-mediated retrograde trafficking of mitochondria was affected in RMS cells. Dynein is a large protein complex composed of dynein heavy chains (DHCs), intermediate chains (DICs), light intermediate chains (DLICs), and light chains (DLCs). The DHC contains the microtubule-binding domain and the AAA motor domain responsible for mitochondrial transport toward the minus-end of the microtubule. To determine whether dynein contributes to the perinuclear distribution of mitochondria in RMS cells, we assessed the levels of dynein subunits by western blotting. The levels of DHC, DIC, and DLIC were significantly higher in RH30 cells compared to both C2C12 and CHQ controls (Figure 5A).

Glycogen synthase kinase 3β (GSK3β) has been shown to regulate dynein activity.^24^ Activated-GSK3β phosphorylates DIC at Ser87/Ser88, which decreases the interaction between DIC and the dynein regulator NDEL1, thereby reducing dynein-based transport.^24^ Because the phosphorylated form pGSK3β(Ser9) is inactive, we determined the ratio of pGSK3β(Ser9) to total GSK3β as a measure of GSK3β activity. We found that the relative level of pGSK3β(Ser9)/GSK3β was higher in RD and RH30 cells than in CHQ and C2C12 cells (Figure 5B), indicating that GSK3β activity was reduced, which implies higher dynein activity. In addition, we previously reported that the relative level of PIP3 on the plasma membrane is higher in RD cells than in C2C12 cells and that overexpression of PTEN in RD cells decreases the level of PIP3 and significantly rescues myoblast fusion.^3^ Thus, the levels of PTEN and PIP3 downstream AKT pathway in four cell lines were compared. Lower level of PTEN and higher level of pAKT/AKT were shown in RD and RH30 cells compared to C2C12 and CHQ cells (Figure 5B). These results suggest that increased PIP3 in RMS cells activates the mTOR/AKT pathway, leading to AKT-mediated phosphorylation of GSK3β at Ser9, which in turn enhances dynein activity and promotes the retrograde trafficking of mitochondria (Figure 5C).

To evaluate the role of elevated dynein activity in RD and RH30 cells, cells were treated with the dynein inhibitor ciliobrevin D, and its effect on mitochondrial distribution was examined by immunofluorescence microscopy and CI. First, the cell viability of RD and RH30 cells with ciliobrevin D was measured. As shown in the Figure 5D, ciliobrevin D, at the indicated concentration range, showed no obvious toxicity to either RD or RH30 cells. The effect of ciliobrevin D on mitochondrial distribution in RD and RH30 cells was subsequently determined. As shown in Figure 5E, ciliobrevin D treatment shifted the mitochondrial distribution from the perinuclear region to the periphery in both RD and RH30 cells. Consistently, mitochondrial CI values decreased in ciliobrevin D-treated cells compared to untreated controls, suggesting that inhibiting retrograde mitochondrial trafficking can partially reverse the perinuclear mitochondrial aggregation observed in RMS cells.

### Inhibiting dynein modulates mitochondrial distribution and myogenic differentiation of RD cells and RH30 cells

**Figure 6:**
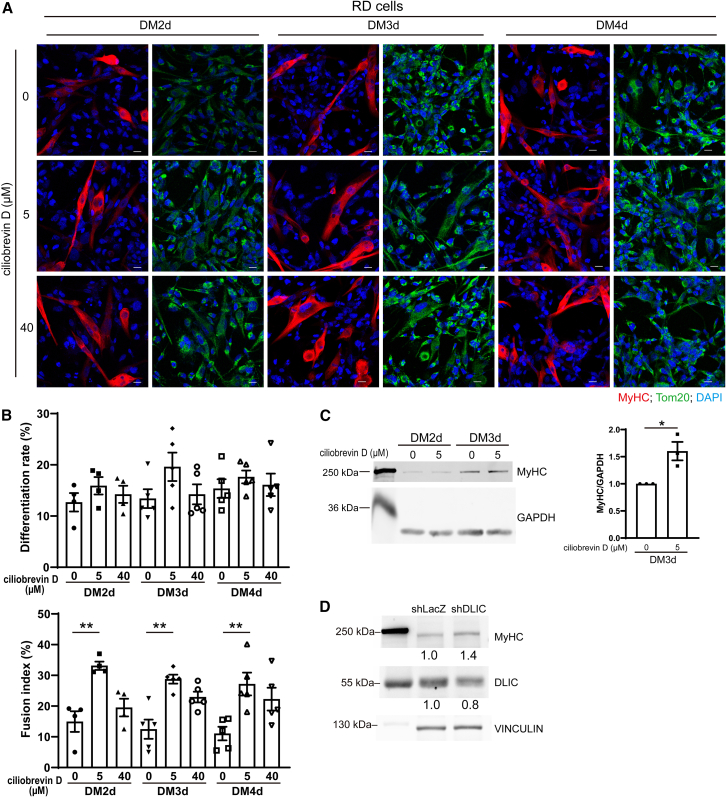
Ciliobrevin D treatment enhances myogenesis in RD cells (A) RD cells were seeded in 12-well plates at 90% confluency and were treated with ciliobrevin D (0, 5, and 40 μM) daily on GM-DM4d. Immunofluorescence staining was performed with anti-MyHC (red) and anti-Tom20 (green) antibodies on DM2d-DM4d. Images were taken on an LSM-800 confocal microscope. Scale bars, 20 μm. (B) Differentiation rate and fusion index of RD cells. Data are presented as mean ± SEM; ∗∗*p* < 0.01, one-way ANOVA. (C) RD cells were treated with or without 5 μM ciliobrevin D till DM3d. Protein levels of MyHC were determined via western blotting and normalized to GAPDH. Data are presented as mean ± SEM; ∗*p* < 0.05, paired *t* test. (D) RD cells were transfected with shLacZ or shDLIC for 48 h and followed by second transfection in differentiation medium for another 72 h. Cell lysate were collected on DM3d. Protein level of MyHC and DLIC were detected by immunoblotting. Protein levels were normalized to VINCULIN.

Given the reduced mitochondrial CI in MyHC^+^ cells (Figure 3) and the observation that ciliobrevin D decreased the mitochondrial CI in both eRMS-derived RD and aRMS-derived RH30 cells (Figure 5E), we next examined whether ciliobrevin D treatment would improve myogenic differentiation of RD cells and RH30 cells. RD cells treated with ciliobrevin D were then induced differentiation. Figure 6A showed that MyHC^+^ (red) cells were relatively elongated due to myoblast differentiation and fusion, and Tom20 in MyHC^+^ cells distributed throughout cells. With the treatment of ciliobrevin D, more MyHC^+^ RD cells were observed indicating enhanced differentiation. The quantified results were shown in Figure 6B top image, which RD cells exhibited a trend of increased differentiation rate with the treatment of 5 μM ciliobrevin D compared to untreated cells on DM2d to DM4d. The myoblast fusion index, calculated as the percentage of cells containing 3 or more nuclei during the formation of myotube, was significantly higher on DM2d to DM4d after 5 μM ciliobrevin D treatment (Figure 6B, bottom). Besides, the protein level of MyHC was increased in RD cells on DM3d with 5 μM ciliobrevin D treatment (Figure 6C). These results indicated that ciliobrevin D treatment can enhance myogenic differentiation of RD cells. Previous studies have established that ciliobrevin D significantly disrupts the Sonic Hedgehog (Shh) signaling pathway.^25^ During skeletal muscle development, Shh modulates myogenic proliferation and differentiation in myogenic cells, and its dynamic regulation is closely linked to myogenic progression through myogenic regulatory factors such as MyoD and Myf-5.^26^^,^^27^^,^^28^ To further confirm the disruption of dynein-mediated mitochondrial transport remain a primary driver of the observed mitochondrial distribution and minimize concerns regarding pharmacological off-target effects, we performed short hairpin RNA (shRNA)-mediated knockdown of DLIC. As shown in Figure 6D, knockdown of DLIC led to an increase of MyHC protein levels in RD cells compared to the shLacZ control.

**Figure 7:**
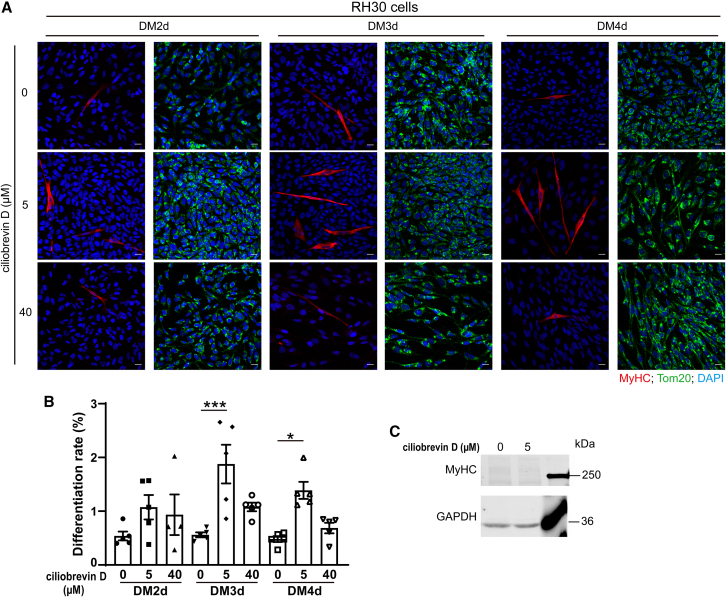
Treatment with ciliobrevin D enhances differentiation in RH30 cells (A) RH30 cells were seeded in 12-well plates at 90% confluency and were treated with ciliobrevin D (0, 5, and 40 μM) daily on GM-DM4d. Immunofluorescence staining was performed with anti-MyHC (red) and anti-Tom20 (green) antibodies on DM2d-DM4d. Images were taken on an LSM-800 confocal microscope. Scale bars, 20 μm. (B) Differentiation rate of RH30 cells. Data are presented as mean ± SEM; ∗*p* < 0.05, ∗∗∗*p* < 0.001, one-way ANOVA. (C) RH30 cells were treated with or without 5 μM ciliobrevin D till DM3d. Protein levels of MyHC were determined via western blotting.

**Figure 8:**
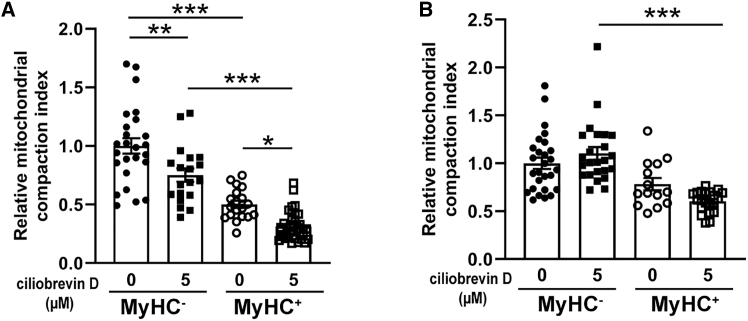
Lower mitochondrial CI in differentiated RD and RH30 cells with ciliobrevin D treatment RD cells (A) and RH30 cells (B) were seeded in 12-well plates at 90% confluency and were treated with ciliobrevin D (0, 5 μM) daily followed by immunofluorescence staining with MyHC and Tom20 antibodies on DM3d. Quantification of mitochondrial CI of MyHC^+^ and MyHC^−^ cells using ImageJ. The CI values were normalized to the average CI value of MyHC^−^ cells without ciliobrevin D. Data are presented as mean ± SEM; ∗*p* < 0.05, ∗∗*p* < 0.01, ∗∗∗*p* < 0.001, one-way ANOVA (*n* = 14–38).

Similarly, the effect of ciliobrevin D treatment on differentiation of RH30 cells was determined. Similar to RD cells, MyHC^+^ RH30 cells treated with ciliobrevin D displayed an elongated morphology with mitochondria distributed throughout the cell, and the number of MyHC^+^ cells increased (Figure 7A). The differentiation rate of RH30 cells was significantly higher on DM3d and DM4d after 5 μM ciliobrevin D treatment (Figure 7B). The fusion index of RH30 cells was 0 with or without ciliobrevin D treatment and the level of MyHC was extremely low in RH30 cells (Figure 7C) The treatment of 40 μM ciliobrevin D did not increase its effect compared to 5 μM, possibly because this high dosage exerts additional effects on transport of other cargoes such as lipid droplets, endosomes, peroxisomes, and RNPs.^29^ These results suggest that treatment with ciliobrevin D reduced mitochondrial CI during the growth phase (Figure 5E) and promoted myogenic differentiation in RD and RH30 cells (Figures 6 and 7). To examine the effect of ciliobrevin D on mitochondrial distribution during differentiation, we assessed the mitochondrial CI

in cells treated with ciliobrevin D on DM3d. In RD cells, the mitochondrial CI was lower with ciliobrevin D treatment in both MyHC^−^ and MyHC^+^ cells compared to no treatment cells. In line with these results, the mitochondrial CI were lower in MyHC^+^ cells compared to MyHC^−^ cells (Figure 8A). In RH30 cells, a modest trend of reduction was observed in MyHC^+^ cells compared to MyHC^−^ cells, this trend became more significant with 5 μM ciliobrevin D treatment (Figure 8B). Together, these results indicated that inhibiting dynein activity modulated mitochondrial distribution, expression of MyHC, myogenic differentiation, and myoblast fusion process.

**Figure 9:**
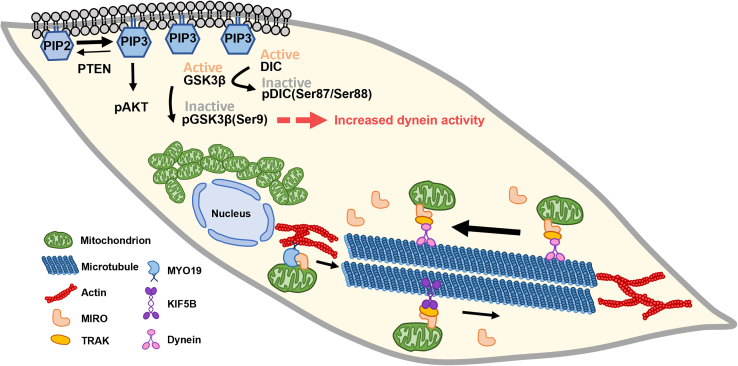
The mechanisms underlie perinuclear aggregation of swollen mitochondria in RD cells The enrichment of PIP3 resulting from low PTEN expression leads to GSK3β phosphorylation via the mTORC2/pAKT pathway. pGSK3β is an inactive form that results in reduced phosphorylation of DIC and thus increased dynein activity. The high level of dynein activity combined with a low level of MYO19 and KIF5B contribute to the perinuclear aggregation of mitochondria in RMS cells.

In summary, the findings of this study reveal a link between mitochondrial distribution and myogenic differentiation. Using human RMS-derived cells, we demonstrated that a reduction in MYO19-mediated anterograde local transport of mitochondria combined with an increase in dynein-mediated retrograde transport resulted in perinuclear clustering of mitochondria (Figure 9). This dysregulation is likely a result of a PTEN deficiency and lead to myogenic differentiation defect of RMS cells.

## Discussion

Mitochondrial morphology and transport are intricately interconnected processes regulated by a dynamic balance of mitochondrial fusion and fission as well as by actin- and microtubule-dependent transport. Findings from this study suggest that an efficient process of short-range, actin-dependent transport as well as long-range, microtubule-based transport is responsible for the cellular distribution of mitochondria and that this distribution governs the success of myoblast fusion during myogenesis. Elongated/fused mitochondria fit well on microtubules structurally and thus can be efficiently transported along microtubules to the cell periphery. In contrast, fragmented and donut-shaped mitochondria tend to aggregate closer to the nucleus, suggesting limited movement via actin around proximal locales. In addition, PIP3-GSK3β-dynein regulation promoted retrograde transport of mitochondria from the cell periphery, exacerbating the clustering of mitochondria.

Mitochondrial morphology and distribution are also linked to the relative cellular abundance of phosphatidylinositol-4-phosphate (PI(4)P).^30^^,^^31^^,^^32^ PI(4)P enrichment at the plasma membrane of the yeast bud results in formation of mitochondria-endoplasmic reticulum-plasma membrane contact sites and redistribution of mitochondria.^31^ In phosphoinositide metabolism, PI(4)P is generated via the dephosphorylation of phosphatidylinositol (3,4)-bisphosphate by PTEN. Notably, PTEN deficiency in RD and RH30 cells were demonstrated in Figure 5B. It is possible that this PTEN deficiency leads to a decreased level of PI(4)P, contributing to the abnormal mitochondrial distribution observed in RMS cells.

It is possible that severe actin bundle defects found in RD cells (Figure 4B) may stem from the oncogenic reprogramming characteristic of RMS. Specifically, recent studies have identified Advillin (AVIL), a gelsolin/villin family protein, as being highly overexpressed in RMS.^33^^,^^34^ AVIL is a potent actin-binding and remodeling protein. AVIL overexpression is known to disrupt normal actin architecture to favor pro-tumorigenic traits like increased motility. Therefore, the observed actin bundle abnormalities in RD cells may result from elevated AVIL levels.

To investigate the genetic alteration that may link to the results of this study, we analyzed the differences in DNA methylation levels between healthy individuals and sarcoma patients. We obtained DNA methylomes of healthy individuals from the Taiwan Biobank (project #TWBR10707-04). Infinium HumanMethylation450 (450K) BeadChip array datasets of sarcoma patients were obtained from The Cancer Genome Atlas-Sarcoma (TCGA-SARC) project on the Genomic Data Commons (GDC) Data Portal (US National Cancer Institute). Figure S1A indicated that the methylation sites of sarcoma patients present significantly different clusters from the control group of healthy individuals. The top 50 genes enriched by differential methylation positions were shown in Figure S1B. Among the 50 most methylation position-enriched genes, protein tyrosine phosphatase receptor type N2 (PTPRN2) has the most DNA methylation positions with more hypomethylated positions, which may lead higher gene expression of PTPRN2. Upregulated PTPRN2 has been reported in breast cancer.^35^ Immature proPTPRN2 interacts with the tumor necrosis factor (TNF) receptor-associated factors (TRAF2) complex and inhibit phosphorylation of TRAF2.^36^ In addition, TRAF2 can induce ubiquitination of Gβl, a known activator of AKT, and further downregulate AKT phosphorylation.^37^ These findings imply that hypomethylated PTPRN2 in sarcoma patients may induce AKT activation in consistent with the results in Figure 5B.

In summary, our results demonstrate that reduced MYO19-dependent anterograde mitochondrial transport combined with elevated dynein-dependent retrograde mitochondrial transport leads to the perinuclear clustering of mitochondria in RMS cells. Increasing MYO19 levels partially rescued this aberrant mitochondrial distribution. Moreover, dynein inhibition restores both mitochondrial distribution and myogenic differentiation in RD and RH30 cells.

### Limitations of the study

Although this study identified a consistent perinuclear mitochondrial distribution across different RMS-derived cell lines, some limitations remain. First, while these findings are robust in *in vitro* models, further validation using primary human RMS tissue sample is necessary to conform whether this spatial organization is a conversed clinical characteristic of RMS. Additionally, while we demonstrated that DLIC knockdown enhances the myogenic capacity of RMS cells, the functional impact of DLIC-mediated mitochondrial positioning on tumorigenesis was not fully explored. Future research using *in vivo* animal models is required to determine how this mitochondrial distribution influences tumor progression and malignancy.

## Resource availability

### Lead contact

Requests for further information and resources should be directed to and will be fulfilled by the lead contact, Linyi Chen (lchen@life.nthu.edu.tw).

### Materials availability

MYO19 plasmid generated in this study will be made available on request but we may require a payment or a completed materials transfer agreement if there is potential for commercial application.

### Data and code availability

• The methylation datasets of healthy individuals used in this study were obtained from the Taiwan Biobank (https://www.twbiobank.org.tw/) and the data are available upon reasonable request and with permission from the Taiwan Biobank, subject to relevant ethical and regulatory approvals. The methylation datasets of sarcoma patients used in this study were publicly available data obtained from TCGA-SARC project on the GDC Data Portal (https://portal.gdc.cancer.gov/projects/TCGA-SARC).
• This paper does not report original code.
• Any additional information required to reanalyze the data reported in this paper is available from the lead contact upon request.

## Acknowledgments

This study was supported by funding from the 10.13039/100020595National Science and Technology Council, Taiwan (grant # NSTC 113-2320-B-007-006-MY3) and from the 10.13039/501100005057National Tsing Hua University, Taiwan (grant # 114QF001E1). We thank the Center of Research in Myology (Paris, France) for providing human CHQ myoblasts and Dr. Lan Bao at the Center for Excellence in Molecular Cell Science (Shanghai Institute of Biochemistry and Cell Biology, Chinese Academy of Sciences) for providing the *KIF5B* constructs. We thank Kuo-Chin Chen for conducting Taiwan Biobank data analysis. We are grateful for the support from the confocal imaging core in National Tsing Hua University, Taiwan, which is sponsored by the 10.13039/100020595National Science and Technology Council (NSTC 114-2740-M-007-001) and instrumentation center in 10.13039/501100005057National Tsing Hua University for Hitachi HT-7700 in TEM analysis. We thank the Academia Sinica Biological Electron Microscopy Core Facility for EM technical support and Dr. Cheng-Fu Kao for technical advice.

## Author contributions

T.L. Ke, conceptualization, data curation, validation, investigation, methodology, and writing – review and editing; L. Chen, conceptualization, investigation, methodology, funding, and writing – review and editing.

## Declaration of interests

The authors declare no competing interests.

## Declaration of generative AI and AI-assisted technologies in the writing process

During the preparation of this work, the authors used Gemini in order to improve the readability and language of the manuscript. After using this tool, the authors reviewed and edited the content as needed and take full responsibility for the content of the publication.

## STAR★Methods

### Key resources table

**Table:** 

REAGENT or RESOURCE	SOURCE	IDENTIFIER
**Antibodies**		

Anti-Tom20	Santa Cruz Biotechnology	sc-11415; RRID: AB_2207533
Anti-KIF5B	Santa Cruz Biotechnology	sc-133184; RRID: AB_2132389
Anti-Tubulin	GeneTex	GTX628802; RRID: AB_2716636
Anti-DLIC	GeneTex	GTX120114; RRID: AB_10720534
Anti-GSK3β	GeneTex	GTX635816; RRID: AB_2888582
Anti-GAPDH	GeneTex	GTX627408; RRID: AB_11174761
Anti-PTEN	GeneTex	GTX101025; RRID: AB_1241223
Anti-MYO19	Novus Biologicals	NBP2-56073; RRID: AB_3341237
Anti-MIRO2	Protientech	11237-1; RRID: AB_2179539
Anti-DHC	Protientech	12345-1; RRID: AB_2261765
Anti-DIC	Protientech	13808-1; RRID: AB_2093492
Anti-pGSK3β	Cell Signaling Technology	8566; RRID: AB_10860069
Anti-AKT	Cell Signaling Technology	9272; RRID: AB_329827
Anti-pAKT	Cell Signaling Technology	4051; RRID: AB_331158
Anti-MIRO1	ABclonal	A22469
Anti-MyHC	Developmental Studies Hybridoma Bank	MF20; RRID: AB_2147781
Alexa Fluor 488 goat anti-mouse IgG	Invitrogen	A11001; RRID: AB_2534069
Alexa Fluor 555 goat anti-mouse IgG	Invitrogen	A21422; RRID: AB_2535844
Alexa Fluor 647 chicken anti-rabbit IgG	Invitrogen	A21443; RRID: AB_2535861
Alexa Fluor 647 goat anti-mouse IgG	Invitrogen	A21235; RRID: AB_2535804
Alexa Fluor 488 donkey anti-rabbit IgG	Invitrogen	A21206; RRID: AB_2535792
Alexa Fluor 700 goat anti-mouse IgG	Invitrogen	A21036; RRID: AB_2535707
IRDye 800CW goat anti-rabbit IgG	LI-COR Biosciences	926-32211; RRID: AB_621843

**Chemicals, peptides, and recombinant proteins**		

Dulbecco’s modified Eagle medium (DMEM)	Gibco	12100046
Antibiotic-antimycotic (AA)	Gibco	15240062
L-glutamine (L-Gln)	Gibco	25030081
Fetal bovine serum (FBS)	Gibco	N/A
Horse serum (HS)	Gibco	16050122
Medium 199	Gibco	41150020
DMEM, high glucose, GlutaMAX Supplement	Gibco	10566016
Gentamicin	Gibco	15750060
Prolong Gold Reagent	Invitrogen	P36930
TRIzol	Invitrogen	15596018
Rhodamine phalloidin	Invitrogen	R415
Tetramethylrhodamine, ethyl ester, perchlorate (TMRE)	Invitrogen	T669
Mitotracker Green	Invitrogen	M7514
DAPI	GeneTex	GTX16206
Digitonin	Sigma-Aldrich	D141
Glutamate	Sigma-Aldrich	G8415
Malate	Sigma-Aldrich	M1000
Oligomycin	Sigma-Aldrich	75351
Carbonyl cyanide 4-(trifluoromethoxy)phenylhydrazone (FCCP)	Sigma-Aldrich	C2920
Rotenone	Sigma-Aldrich	R8875
Antimycin A	Sigma-Aldrich	A8674
Succinate	Sigma-Aldrich	S3674
ADP	Sigma-Aldrich	A2754
N,N,N′,N′-Tetramethyl-*p*-phenylenediamine dihydrochloride (TMPD)	Sigma-Aldrich	T3134
Ascorbate	Sigma-Aldrich	A4034
Bovine serum albumin (BSA)	Sigma-Aldrich	A9647
Ciliobrevin D	Sigma-Aldrich	250401
TransIT®-LT1 Transfection Reagent	Mirus Bio	MIR2300

**Critical commercial assays**		

CellTiter-Glo® luminescent cell viability assay	Promega Corporation	G7570

**Experimental models: Cell lines**		

C2C12 cells	Bioresource Collection and Research Center	60083
RD cells	Bioresource Collection and Research Center	60113
RH30 cells	Bioresource Collection and Research Center	60420
CHQ cells	Provided by Dr. Vincent Mouly (Center of Research in Myology, Sorbonne University, Paris, France)	N/A

**Oligonucleotides**		

hMYO19_Q_F	This study	5′-GGGTGAATCCTGTGACACTAGA-3′
hMYO19_Q_R	This study	5′-GCCAGCATTGGTGTAGAATGT-3′
mMYO19_Q_F	This study	5′-CTCAAGGGAGACCTAAGGGAG-3′
mMYO19_Q_R	This study	5′-CTGTTTCCAGTGTCACGGGAT-3′
hKIF5B_Q_F	This study	5′-GAGTTAGCAGCATGTCAGCTTCG-3′
hKIF5B_Q_R	This study	5′-GCATCGACAGATTCCTCCAACTG-3′
mKIF5B_Q_F	This study	5′-GCGAGATGAAGTGGAGGCAAAG-3′
mKIF5B_Q_R	This study	5′-CTCTTGGTCTGTAGCCTTCAGC-3′
hGAPDH_Q_F	This study	5′-TCAAGGCTGAGAACGGGAAG-3′
hGAPDH_Q_R	This study	5′-CGCCCCACTTGATTTTGGAG-3′
mGAPDH_Q_F	This study	5′-ATGTTTGTGATGGGTGTGAA-3′
mGAPDH_Q_R	This study	5′-ATGCCAAAGTTGTCATGGAT-3′

**Recombinant DNA**		

GFP-MYO19	Addgene	134987
MYO19-GFP	Addgen	134988
mito-meGFP	Addgen	172481
mCherry-Fis1	Addgen	182580
MYO19	This study	N/A
KIF5B-GFP	Provided by Dr. Lan Bao (Center for Excellence in Molecular Cell Science, Shanghai Institute of Biochemistry and Cell Biology, Chinese Academy of Sciences)	N/A
KIF5B^G234C^-GFP	Provided by Dr. Lan Bao (Center for Excellence in Molecular Cell Science, Shanghai Institute of Biochemistry and Cell Biology, Chinese Academy of Sciences)	N/A
KIF5B^1-330^-GFP	Provided by Dr. Lan Bao (Center for Excellence in Molecular Cell Science, Shanghai Institute of Biochemistry and Cell Biology, Chinese Academy of Sciences)	N/A

**Software and algorithms**		

ImageJ (version 1.53t)	NIH (National Institutes of Health)	RRID:SCR_003070
ZEISS ZEN Microscopy Software blue edition (version 2.6.76)	Carl Zeiss Microscopy	RRID:SCR_013672
CytoExpert (version 2.4.0.28)	Beckman Coulter Life Sciences	RRID:SCR_017217
GraphPad Prism (version 8.0.2)	GraphPad Software	RRID:SCR_002798
DatLab (6.1.0.7)	Oroboros Instruments GmbH	N/A
Image Lab (version 6.1.0)	Bio-Rad	RRID:SCR_014210
StepOne Software (version 2.3)	Life Technologies Corporation	RRID:SCR_014281

### Experimental model and study participant details

C2C12 cells (60083), RD cells (60113) and RH30 cells (60420) were purchased from Bioresource Collection and Research Center (Hsinchu, Taiwan). Human CHQ cells were donated from Dr. Vincent Mouly (Center of Research in Myology, Sorbonne University, Paris, France). Cell lines were authenticated by the providers. Cell lines were tested for mycoplasma contamination and the results were negative. C2C12 cells, RD cells and RH30 cells were cultured in growth medium (DMEM with 1% AA, 1% l-Gln and 10% FBS) and incubated at 37°C in humidified atmosphere containing 5% CO_2_. Differentiation was induced by switching to low-serum medium (DMEM with 1% AA, 1% l-Gln and 2% HS) when cells reached 90% occupancy. CHQ cells were cultured in growth medium (4:1 (v/v) DMEM/GlutaMAX and Medium 199 with 20% FBS and 50 μg/mL gentamicin) and incubated at 37°C in a humidified atmosphere containing 5% CO_2_.

### Method details

#### Plasmid construction and transient transfection

Plasmids GFP-MYO19 (Addgene #134987) and MYO19-GFP (Addgene #134988) were gifts from Martin Bähler.^13^ Plasmid mito-meGFP (Addgene #172481) was a gift from Thomas Schwarz, and mCherry-Fis1 (Addgene #182580) was a gift from Uri Manor.^38^ Plasmid MYO19 was generated by inserting PCR-amplified full-length MYO19 into GFP-MYO19 between the BglII and MfeI sites. The various truncations of MYO19 were constructed using PCR-amplified fragments inserted into GFP-MYO19 between the NheI and MfeI sites. KIF5B-GFP, KIF5B^G234C^-GFP and KIF5B^1-330^-GFP were gifts from Lan Bao (Center for Excellence in Molecular Cell Science, Shanghai Institute of Biochemistry and Cell Biology, Chinese Academy of Sciences, Shanghai, China). Knockdown constructs, shLacZ and shDLIC constructs were purchased from the National RNAi Core Facility of Academia Sinica (Taipei, Taiwan). Cells were transiently transfected with plasmids using TransIT-LT1 Transfection Reagent. After 48 h, transfected cells were used for various assays. For knockdown experiment, RD cells were transfected with shLacZ or shDLIC in culture medium for 48 h and followed by second transfection in differentiation medium for another 72 h. Cell lysate were collected on DM3d.

#### Transmission electron microscope (TEM)

Cells were seeded on the aclar film. Cells were first fixed with 2.5% glutaraldehyde and 1% tannic acid in 0.1 M cacodylate buffer at room temperature for 60 min. Post-fixation was performed using 1% osmium tetroxide and 1.5% potassium ferrocyanide in 0.1 M cacodylate buffer at room temperature for an additional 60 min. Following fixation, samples were dehydrated through a graded ethanol series (15%, 30%, 50%, 75%, 80%, 90%, 95%, and 100%), with each step performed for 10 min, and the 100% ethanol step repeated four times. Dehydrated samples were then infiltrated and embedded in Spurr’s resin according to standard procedures. Ultrathin sections (90 nm) were cut using an ultramicrotome and collected on nickel grids. Sections were subsequently stained with 4% uranyl acetate for 10 min, followed by Reynolds’ lead citrate for 8 min, prior to TEM observation. Images were taken by Hitachi HT7700 high contrast transmission electron microscope.

#### Immunofluorescence staining, image analysis and western blotting

Cells were fixed with 4% paraformaldehyde for 10 min, then permeabilized with 0.1% (w/v) Triton X-100 for 10 min, and incubated in blocking buffer (1% bovine serum albumin in phosphate-buffered saline) for 1 h. Next, cells were incubated with the primary antibody of interest at 4°C overnight, followed by conjugated secondary antibody for 1 h, and mounted in Prolong Gold reagent. Immunofluorescence images were taken using a Zeiss ZEN confocal microscope LSM-800 with Airyscan.

Cell lysates were collected into radioimmunoprecipitation (RIPA) lysis buffer containing protease inhibitors (10 ng/mL aprotinin and 10 ng/mL leupeptin (A + L), 1 mM PMSF) and phosphatase inhibitors (1 mM Na_3_VO_4_), incubated on ice for 10 min, and then centrifuged at 16.2 × *g* for 15 min at 4°C. Equal amounts of protein were resolved by SDS-PAGE. Blots were incubated with primary antibodies at 4°C overnight followed by IRDye-conjugated IgG for 1 h. Signals were detected using ChemiDoc MP Imaging Systems (Bio-Rad, Hercules, California, USA).

#### Real-time semi-quantitative polymerase chain reaction

RNAs were extracted from cells using TRIzol reagent. mRNA (2 μg) was reverse-transcribed to cDNA. Real-time PCR with SYBR Green detection was performed using an ABI PRISM 7500 sequence detection system (Applied Biosystems, Waltham, Massachusetts, USA). Glyceraldehyde-3-phosphate dehydrogenase (GAPDH) was used as a control. Primers used were listed in supplemental information (Table S1).

#### Determination of mitochondrial membrane potential and function

Cells were harvested and incubated in growth medium containing 100 nM TMRE and 100 nM MitoTracker Green at 37°C for 30 min with shaking. Medium was then replaced with phosphate-buffered saline to reduce background signal, and the intensity of TMRE and MitoTracker Green was measured using a CytoFLEX Flow Cytometer (Beckman, Brea, California, USA). CytExpert software was used for data analysis. Data are presented as a ratio of TMRE to MitoTracker Green to normalize membrane potential for mitochondrial mass.

To measure oxygen consumption rates, undifferentiated cells were harvested in growth medium at 6 × 10^5^ cells/mL and added to a 2-mL chamber of the Oxygraph-2k (Oroboros Instruments, Innsbruck, Austria). Addition of 1 μM oligomycin was used to inhibit ATP synthase, stepwise addition of 2 μM FCCP was used to induce a state of maximum respiration up to maximum stimulation of uncoupled respiration, and addition of 1 μM rotenone (complex I activity inhibitor) and 2 μM antimycin A (complex III activity inhibitor) was used to inhibit respiration.

To measure mitochondrial complex activities, undifferentiated cells were harvested in MiR05 buffer at 3.5 × 10^5^ cells/mL and added to a 2-mL chamber of the Oxygraph-2k. Addition of 5 μM digitonin was used to permeabilize cells, stepwise addition of 10 mM glutamate, 2 mM malate and 5 mM ADP was used to induce mitochondrial complex I, and this was followed by addition of 1 μM rotenone (complex I activity inhibitor). Then, 10 mM succinate was added to induce mitochondrial complex II and 2 μM antimycin A to inhibit complex III activity. Finally, addition of 0.4 mM ascorbate and 0.1 mM TMPD was used to induce mitochondrial complex IV activity and addition of NaN_3_ inhibited complex IV activity.

#### Cell viability assays

RD and RH30 cells were cultured in 96-well white plate and ciliobrevin D was added in culture medium with final concentration of 3 nM–60 μM after cells fully attached to the well. After 24 h, CellTiter-Glo reagent (Promega) was added to each well and the luminescent signal was recorded by microplate reader.

#### Taiwan Biobank data and DNA methylation analysis

Methylation datasets of healthy individuals were analyzed using data from the Taiwan Biobank (project #TWBR10707-04). Infinium HumanMethylation450 (450K) BeadChip array datasets of sarcoma patients were obtained from The Cancer Genome Atlas (TCGA-SARC) project on the GDC Data Portal (U.S. National Cancer Institute). All methylation datasets were analyzed using R (4.0.2) and the Chip Analysis Methylation Pipeline (ChAMP) package. The champ.filter function was used to filter the data.

### Quantification and statistical analysis

The relative mitochondrial area was quantified by ImageJ software using the formula (mitochondrial area/cellular area) × 100%. The mitochondrial compaction index was quantified by ImageJ using the formula [2π × (area/π)ˆ(½)]/perimeter.^39^ The intensity histogram of images was quantified using the ZEN blue software (Zeiss). The Differentiation rate was calculated by number of nuclei in MyHC^+^ cells)/total number of nuclei. Cells containing at least three nuclei were considered to be myotubes. The fusion index (%) was calculated as the number of nuclei (≥3) in myotubes divided by the total number of nuclei in MyHC^+^ cells. All data are presented as mean ± S.E.M. of data obtained from at least three independent experiments. Statistical analysis was carried out using one-way analysis of variance (ANOVA) and statistical significance symbols were defined as ∗*p* < 0.05, ∗∗*p* < 0.01, and ∗∗∗*p* < 0.001. Statistical significance symbols were defined as #*p* < 0.05 using unpaired *t* test.
