# Supplementary material for: High level dynein impairs mitochondrial distribution and differentiation of rhabdomyosarcoma cells
Source: iScience. 2026 May 22;29(6):116057. doi: 10.1016/j.isci.2026.116057 (PMC13224007; doi:10.1016/j.isci.2026.116057)
Supplement: Document S1. Figure S1 and Table S1 [file mmc1.pdf]

iScience, Volume *29*

Supplemental information

High level dynein impairs mitochondrial distribution and differentiation of rhabdomyosarcoma cells

Ting-Ling Ke and Linyi Chen

Document S1

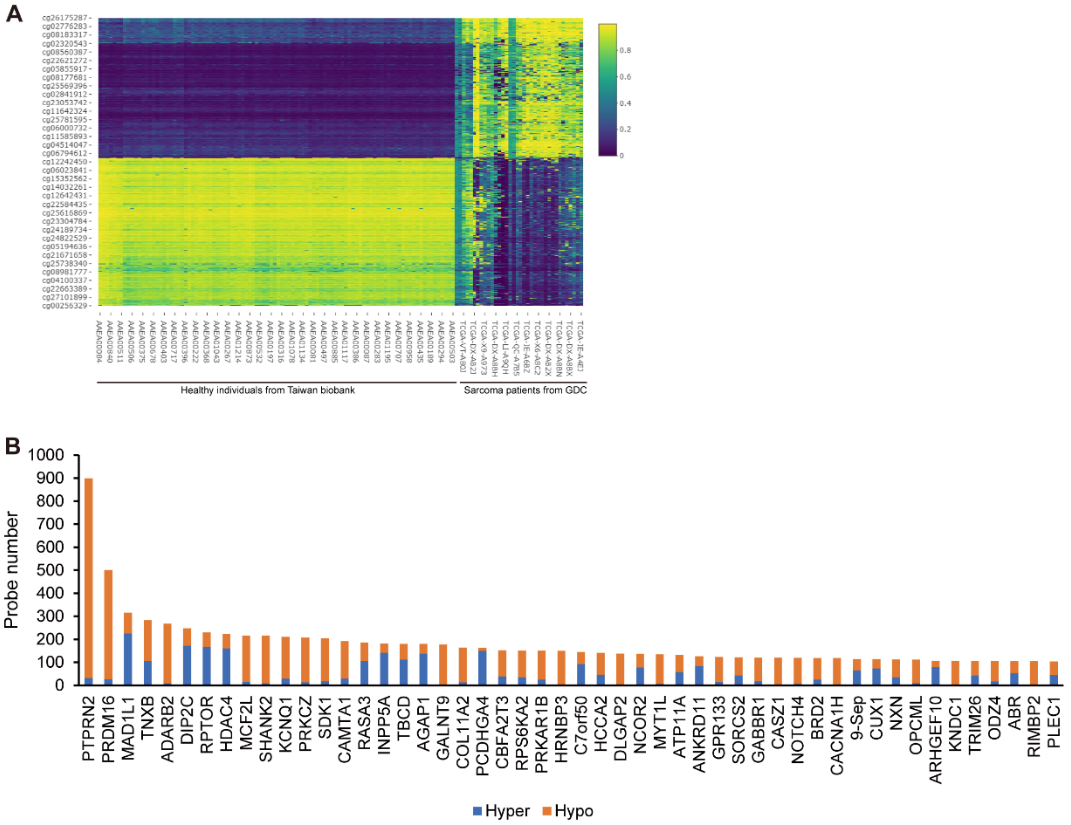

Figure S1. Hypomethylation of PTPRN2 in sarcoma patients compared to healthy individuals. (A) Comparison of methylation datasets between sarcoma patients (TCGA-SARC, GDC Portal Database) and healthy individuals (Taiwan Biobank). The heatmap shows all detected CpG positions of healthy individuals and sarcoma patients through hierarchical clustering of rows and columns. (B) The box chart shows the top 50 methylation-enriched genes.

Table S1. Primer list of qPCR

Primer name	Sequence
hMYO19_Q_F	5'-GGGTGAATCCTGTGACACTAGA-3'
hMYO19_Q_R	5'-GCCAGCATTGGTGTAGAATGT-3'
mMYO19_Q_F	5'-CTCAAGGGAGACCTAAGGGAG-3'
mMYO19_Q_R	5'-CTGTTTCCAGTGTCACGGGAT-3'
hKIF5B_Q_F	5'-GAGTTAGCAGCATGTCAGCTTCG-3'
hKIF5B_Q_R	5'-GCATCGACAGATTCCTCCAACTG-3'
mKIF5B_Q_F	5'-GCGAGATGAAGTGGAGGCAAAG-3'
mKIF5B_Q_R	5'-CTCTTGGTCTGTAGCCTTCAGC-3'
hGAPDH_Q_F	5'-TCAAGGCTGAGAACGGGAAG-3'
hGAPDH_Q_R	5'-CGCCCCACTTGATTTTGGAG-3'
mGAPDH_Q_F	5'-ATGTTTGTGATGGGTGTGAA-3'
mGAPDH_Q_R	5'-ATGCCAAAGTTGTCATGGAT-3'
